# Multiple jejuno-jejunal fistulae of uncertain origin: a case report

**DOI:** 10.1186/1752-1947-1-145

**Published:** 2007-11-23

**Authors:** Iraklis E Katsoulis, Shirley Y Chan, Emin A Carapeti

**Affiliations:** 1 Department of Surgery, Guy's and St Thomas' NHS Foundation Trust, London, UK

## Abstract

A 43 year-old male patient presented with small bowel obstruction while being treated for cervical tuberculous lymphadenopathy. Laparotomy revealed multiple adhesions and multiple jejuno-jejunal fistulae. Absence of previous abdominal surgery or other abdominal insult favoured an 'idiopathic' origin of these unusual lesions, although treated tuberculosis may have been the underlying cause. To the best of our knowledge this intestinal condition has never previously been reported in the medical literature.

## Introduction

Adhesions are by far the commonest cause of small bowel obstruction. Other causes include hernias, neoplasms, inflammatory causes, mesenteric vascular occlusion and intussusception. There are also reports of various unusual causes [1].

## Case presentation

**Figure 1:**
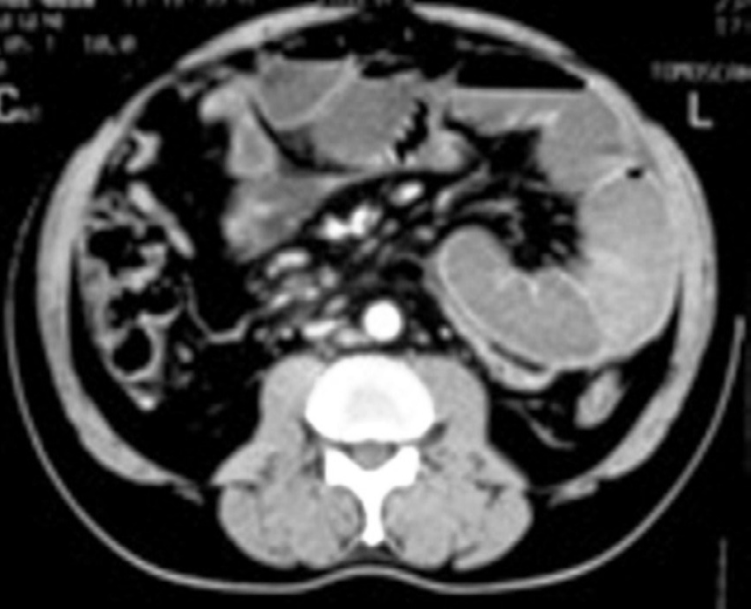
Abdominal CT showing small bowel obstruction.

**Figure 2:**
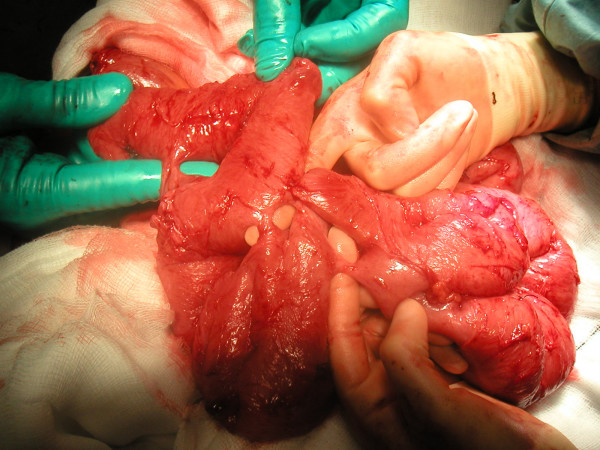
Multiple fistulae between loops of jejunum.

A 43 year-old Ethiopian male presented with a 3 day history of generalized colicky abdominal pain and vomiting. The patient was on the third week of anti-tuberculous therapy for cervical tuberculous lymphadenopathy. There was no previous history of abdominal surgery or pulmonary tuberculosis (TB) and the patient's plain chest x-ray was normal. Physical examination was consistent with an intestinal obstruction. High resolution computed tomography (CT) of the abdomen was performed with oral water soluble contrast and this showed a distal small bowel obstruction with marked dilatation and no evidence of a focal mass lesion or free fluid (figure 1). In view of the persistence of the obstruction a laparotomy was undertaken. At operation multiple generalized peritoneal adhesions were found. No tuberculomas, ascites or other acute or chronic inflammatory changes were identified. The small bowel was dilated proximal to the terminal ileum, which was collapsed. This was due to multiple adhesions with no single discrete obstructing lesion identified at that level. The sigmoid colon was noted to be long and redundant but otherwise normal. After meticulous and laborious adhesiolysis, multiple (more than 15) jejuno-jejunal fistulae were found (figure 2). All fistulae were divided and the resulting enterotomies on both luminal aspects repaired, thus releasing the small bowel along its whole length.

Recovery of bowel motility and absorption was slow and the patient required parenteral nutrition for a period of 10 days. He was discharged home on the 20^th ^postoperative day. Three months after discharge the patient had no residual abdominal symptoms and was well nourished.

## Discussion

Clinical presentation of this patient in combination with his history of cervical tuberculous lymphadenopathy raised suspicion of abdominal TB. However, CT showed small bowel obstruction without appearances typical of TB [2]. Furthermore, laparotomy revealed extensive adhesions and multiple jejuno-jejunal fistulae and there was no evidence of tuberculomas, ascites or other acute or chronic inflammatory changes. Adhesions were the apparent cause of intestinal obstruction. The aetiology of these was not however clear, nor that of the fistulae between the jejunal loops. Absence of previous abdominal surgery or other abdominal insult favoured an 'idiopathic' origin of these lesions, although treated TB may have been the underlying cause. We do not know if it was necessary to divide all or any of the fistulous bridges between the jejunal loops. However, in view of the rarity of the findings and lack of previous experience with such a case, it was felt that leaving these fistulae intact might result in further obstructive episodes due to internal herniation and incarceration.

## Conclusion

Regardless of their underlying aetiology, jejuno-jejunal fistulae are a very unusual pathology which can be associated with intestinal obstruction. To the best of our knowledge there are no previous reports of this condition in the literature.

## Competing interests

The author(s) declare that they have no competing interests.

## Authors' contributions

IEK prepared the manuscript and performed the literature search. SYC contributed to the illustration preparation. EAC critically reviewed the manuscript.

## Consent

Written informed patient consent was obtained for publication.
